# Increased risk of lower limb osteoarthritis among former professional soccer (football) players

**DOI:** 10.1093/occmed/kqad132

**Published:** 2023-12-09

**Authors:** E R Russell, S J Spencer, C M Atherton, D M Lyall, D F Mackay, K Stewart, J A MacLean, J P Pell, W Stewart

**Affiliations:** School of Psychology and Neuroscience, University of Glasgow, Glasgow G12 8QQ, UK; Department of Orthopaedic Surgery, Queen Elizabeth University Hospital, Glasgow, G51 4TF, UK; Department of Trauma and Orthopaedics, Queen Elizabeth University Hospital, Glasgow, G51 4TF, UK; School of Health and Wellbeing, University of Glasgow, Glasgow G12 8TB, UK; School of Health and Wellbeing, University of Glasgow, Glasgow G12 8TB, UK; School of Cardiovascular and Metabolic Health, University of Glasgow, Glasgow G12 8TA, UK; Hampden Sports Clinic, Hampden Stadium, Glasgow G42 9ED, UK; School of Cardiovascular and Metabolic Health, University of Glasgow, Glasgow G12 8TA, UK; Hampden Sports Clinic, Hampden Stadium, Glasgow G42 9ED, UK; School of Health and Wellbeing, University of Glasgow, Glasgow G12 8TB, UK; School of Psychology and Neuroscience, University of Glasgow, Glasgow G12 8QQ, UK; Department of Neuropathology, Queen Elizabeth University Hospital, Glasgow G51 4TF, UK

## Abstract

**Background:**

Soccer is a high-speed contact sport with risk of injury. Despite long-standing concern, evidence to date remains inconsistent as to the association between playing professional-level soccer and lifelong musculoskeletal consequences.

**Aims:**

The objectives were to assess risk of osteoarthritis in former professional soccer players compared to matched general population controls, and subsequently assess associated musculoskeletal disorders which may contribute to, or result from, osteoarthritis—specifically meniscal injury and joint replacement.

**Methods:**

We conducted a retrospective cohort study using national electronic health records (EHRs) on a cohort of 7676 former professional soccer players aged 40 or over at recruitment, matched on year of birth, sex (all male) and socio-economic status with 23 028 general population controls. Outcomes of interest were obtained by utilizing individual-level record linkage to EHRs from general hospital inpatient and day-case admissions.

**Results:**

Compared to controls, former soccer players showed a greater risk of hospital admission for osteoarthritis (hazard ratio [HR] 3.01; 95% confidence interval [CI] 2.80–3.25; *P* < 0.001). This increased risk appeared age dependant, normalizing over age 80 years and reflective of increased risk of lower limb osteoarthritis. Further, risk of hospital admissions for meniscal injury (HR 2.73; 95% CI 2.42–3.08; *P* < 0.001) and joint replacement (HR 2.82; 95% CI 2.23–3.57; *P* < 0.001) were greater among former soccer players.

**Conclusions:**

We report an increased risk of lower limb osteoarthritis in former soccer players when compared with matched population controls. The results of this research add data in support of lower limb osteoarthritis among former soccer players representing a potential industrial injury.

## Teaser

By utilizing national electronic health records on a large cohort of former professional soccer players, we show an age-associated increased risk of osteoarthritis in soccer players when compared with matched population controls, a risk which was mainly attributed to lower limb osteoarthritis. These results add to the evidence base relating to risk of musculoskeletal issues in soccer players, adding to data in support of lower limb osteoarthritis representing a potential industrial injury in these former athletes.

**Key learning points**

**What is already known about this subject:**

- Injuries are common in sporting athletes, especially in those who play at a professional level.
- There is emerging evidence to highlight increased musculoskeletal issues in retired elite-level sporting athletes.

**What this study adds:**

- Evidence to date regarding musculoskeletal issues, particularly osteoarthritis, in former soccer players remains limited.
- This study reports on age-associated increase in risk of osteoarthritis, indicated in hospital admission data, in former soccer players when compared with matched population controls, thus contributing to the literature in this field.

**What impact this may have on practice or policy:**

- These data add to the evidence base relating to risk of musculoskeletal issues in soccer players, and to data in support of lower limb osteoarthritis representing a potential industrial injury in these former athletes.

## Introduction

Soccer is a high-speed contact sport, which is not without risk of injury. Professional soccer players are reported to suffer 710 injuries per 100 000 hours of play, in comparison to an average 0.36 injuries per 100 000 working hours in other UK employees [1–3]. Specifically, injuries of the lower limb are frequent during soccer matches, due to the high-speed, competitive nature of the game [4]. Despite long-standing concerns regarding incidence of musculoskeletal injuries and their potential long-term consequences, evidence to date remains inconsistent as to the association between a career in professional-level soccer and lifelong musculoskeletal consequences [5].

Injuries can have long-term impacts, with approximately 47% of players forced to retire from professional-level soccer as a result of an acute or chronic injury [1], and health-related quality of life reported to be lower in retired players with osteoarthritis (OA) [6]. Risk of arthritis has also been noted in other contact sports, with rates of arthritis in former National Football League players around three times higher than in the US male general population [7], and professional American footballers reportedly 10 times more likely to undergo total knee or hip arthroplasties than men in the US general population [8]. In rugby, arthritis has been reported as over 2-fold greater in former elite players, when compared with non-contact athletes [9]. However, previous studies in soccer have methodological limitations, including modest sample sizes and failure to identify appropriately matched control populations. The literature to date has been reported as providing low-quality evidence of increased joint issues, and OA in former professional soccer players [10].

OA is a degenerative joint disease, characterized by cartilage degradation, inflammatory response and reduced mobility. Symptoms include joint pain, swelling and stiffness, which may be aggravated by prolonged periods of both movement and rest [11]. In severe cases, joint replacement (arthroplasty) may be indicated. Clinically, the knee is the greatest affected, with an estimated 85% of OA burden relating to the knee [12]. There is growing research indicating that OA is more prevalent in individuals who undertake physically demanding occupations which involve excessive and repetitive use of joints [13]. Conversely, provided trauma to the joints is avoided, there is evidence to suggest beneficial effects of moderate exercise on the knee joints, such as reduced pain and disability, and no evidence of accelerated development of OA [14].

The objectives of this study were to assess the risk of hospital admission for arthritis, both rheumatoid arthritis (RA) and OA, in former professional soccer players compared to matched general population controls. Furthermore, OA risk was assessed with regards to professional active career length to test the hypothesis that longer exposure to injury is associated with a greater risk of OA, with career length used as a measure of cumulative exposure. By utilizing hospital inpatient and day-case records from 7676 former professional soccer players, matched with 23 028 population controls, we contribute to the understanding of the association between a career in professional-level soccer and musculoskeletal outcomes.

## Methods

Ethical approval and proportionate governance reviews were provided by the University of Glasgow College of Medical, Veterinary and Life Sciences Ethics Committee (project number 200160147) and National Health Service Scotland’s Public Benefit and Privacy Panel for Health and Social Care (reference 1718-0120), respectively. The complete protocol for ‘Football’s InfluencE on Lifelong health and Dementia risk’ (FIELD) is published elsewhere [15]. The analysis and reporting of this study are consistent with the Strengthening the Reporting of Observational Studies in Epidemiology (STROBE) reporting guidelines [16].

Former professional soccer players were identified from the Record of Pre-war Scottish League Players (v2) [17], and the Record of Post-War Scottish League Players (v6) [18]. Probabilistic matching was used to seed the data on former players with their Community Health Index, a unique identifier that is used on all electronic health records. The Community Health Index database was used to identify controls, with each former soccer player (FSP) matched to three general population controls by sex (all male), year of birth and area-level socio-economic status measured by the Scottish Index of Multiple Deprivation (SIMD), derived from last known postcode of residence. Study inclusion was restricted to individuals 40 years or over as of 31 December 2016. Full details regarding cohort identification and inclusion criteria are published elsewhere, as is information regarding the matching of population controls [15,19].

Individual-level data on FSPs and their matched population controls (MPC) were obtained by interrogation of the Scottish Morbidity Record (SMR) 01 which covers both general/acute inpatient and day-case hospital admissions across Scotland from 1981 onwards. SMR01 contains ICD9/ICD10 codes that were used to ascertain musculoskeletal-related admissions as the primary cause of hospital admission (Table 1). The dataset also records the date of hospital admission, which was used as the date of the incident event. A similar methodology has been used previously in highly successful studies [20–22].

**Table 1.** ICD9/ICD10 codes used for analysis

Outcome	ICD9	ICD10
Osteoarthritis	715	M15 to M19
Upper limb	715·11 to ·14; 715·21 to ·24 & ·31 to ·34 & ·91 to ·94	M18; M19·01 to ·04 & ·11 to ·14 & ·21 to ·24
Lower limb	715·15 to ·17 & ·25 to ·27 & ·35 to ·37 & ·95 to ·97	M16; M17; M19·07 & ·17 & ·27
Rheumatoid arthritis	714	M05; M06; M08
Joint replacement	V43·6	Z96·6
Meniscal injury	717 844	M23 S83

Hospital admissions relating to OA were our primary outcome measure. As a comparison analysis, RA was also investigated. Other musculoskeletal outcomes that have been previously reported as relating to OA were also assessed. Meniscal injury is not uncommon among contact sport athletes, with meniscal tears being among the most common knee injuries in this population. Meniscal tears have been highlighted as potentially both leading to knee OA and resulting from OA. Complete joint replacement has also been highlighted as an outcome following severe OA. RA is an autoimmune inflammatory disorder, which results in damage to the joints and cartilage and can cause pain, swelling and joint stiffness [23].

Professional career length was defined as the length of active professional career and did not include years spent in non-professional soccer. Career length was categorized as short (1–5 years), low–medium (6–10 years), high–medium (11–15 years) and long (16 years or over). Outliers defined as those with a career length greater than 2 standard deviations (SDs) above the mean (mean [SD] 8.6 [6.2]) years) were excluded from these analyses as some may have reflected incorrect recording, such as inclusion of time spent in a managerial or coaching role. Individuals with missing career length information were also excluded from this analysis. For the analysis of player position, FSPs were categorized as either goalkeeper or outfield player. Outfield players were subsequently subdivided into defender, midfielder, forward or, where a player had participated in several field positions, multi-position.

Cox proportional hazards regression was used to model time to first hospital admission for musculoskeletal disease among FSPs referent to controls. Schoenfeld residuals were used to test the assumption of proportional hazards. Where the assumption did not hold, a time-varying model was used to derive hazard ratios (HRs) over different periods of follow-up. Time-varying HRs and 95% confidence intervals (CIs) were calculated using the ‘lincom’ command in Stata software. If no hospital admissions for eligible musculoskeletal diseases occurred, follow-up was censored at either date of death or 31 December 2016, the last date for which records were available. The model was repeated with the sub-group of FSPs stratified by player position and then career duration as the exposure variable. Soccer players were kept matched with their population controls for the analyses of all outcomes.

Age at admission was compared between FSPs and the control group, also subsequently stratified by player position and by career duration, using linear regression with robust standard errors. A likelihood-ratio test was performed to indicate statistical certainty of the relationship between specific variables. All statistical analyses were performed using Stata v16 [24] with statistical significance set at two-sided *P* < 0.05.

## Results

**Figure 1.**
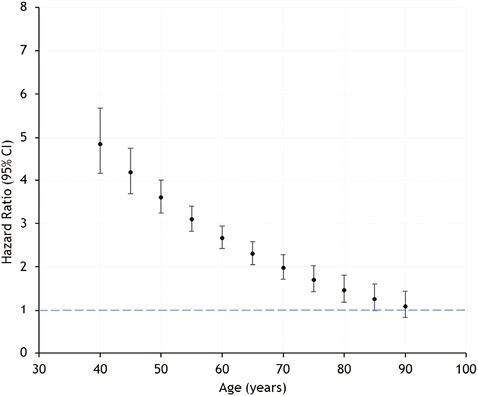
A time-dependent analysis showing osteoarthritis hospital admission dependent on age (years) for former soccer players (FSP) compared to their matched population controls (MPC). Risk of osteoarthritis was greater among FSP up to the age of 80 years, and not significantly different from MPC thereafter.

Compared with MPC, FSPs were more likely to be admitted to hospital for OA in the overall Cox model (HR 3.01; 95% CI 2.80–3.25; *P* < 0.001) (Table 2); however, the proportional hazard assumption was not met, and risk of admission for OA varied between upper and lower limbs. A time-dependent analysis showed that the excess risk among FSPs was highest at age 40–45 years (HR 4.85; 95% CI 4.15–5.68), with the strength of the association then declining until the age 75–80 years (HR 1.47; 95% CI 1.19–1.81); no different to the general population thereafter (Figure 1). While there were no differences between FSPs and MPC in hospital admission for OA of the upper limb (HR 1.54; 95% CI 0.95–2.50; *P* = 0.081), admissions for lower limb OA were greater among FSPs (HR 3.45; 95% CI 3.17–3.76; *P* < 0.001). Again, this latter relationship was age dependent, with hospital admission for lower limb OA greater among FSPs to the age of 85 years; thereafter no different to that of MPC.

**Table 2.** Cox proportional hazard models of hospital admissions for arthritis and joint replacement in former soccer players (FSP) referent to matched population controls (MPC)

Hospital admission	Former soccer players *n*(%)	Matched population controls *n*(%)	Hazard ratio (95% CI)	*P* ^a^
Osteoarthritis^†^	1046 (14)	1107 (5)	3.01 (2.80–3.25)	<0.001
Upper limb			1.54 (0.95–2.50)	0.081
Lower limb^b^			3.45 (3.17–3.76)	<0.001
Rheumatoid arthritis	41 (1)	131 (1)	0.98 (0.72–1.33)	0.902
Joint replacement	171 (2)	181 (1)	2.82 (2.23–3.57)	<0.001
Meniscal injury	355 (5)	416 (2)	2.73 (2.42–3.08)	<0.001

^a^ *P*-values were calculated by the use of a Cox proportional hazards regression.

^b^Osteoarthritis overall, and osteoarthritis of the lower limb did not fulfil the proportional hazards assumption.

Risk of hospital admission for meniscal injury (HR 2.73; 95% CI 2.42–3.08; *P* < 0.001) and joint replacement (HR 2.82; 95% CI 2.23–3.57; *P* < 0.001) were greater in FSPs than in MPC (Table 2). There were no differences in hospital admissions for RA between FSPs and their MPC (HR 0.98; 95% CI 0.72–1.33; *P* = 0.902) (Table 2).

**Figure 2.**
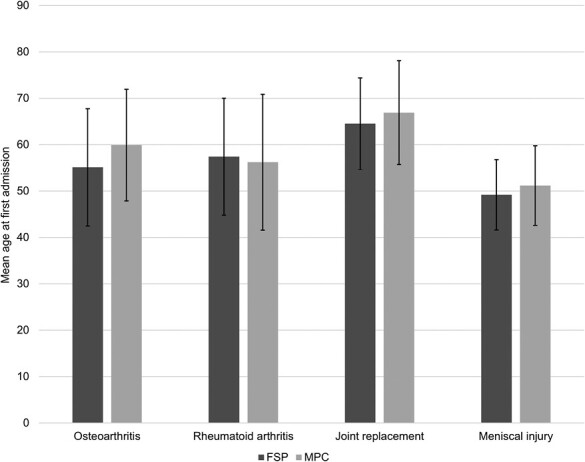
Mean age (SD) at first hospital admission with arthritis or joint replacement. Mean age at first admission was lower in former soccer players (FSP) for osteoarthritis, joint replacement and meniscal injury when compared with matched population controls (MPC). There were no differences in mean age at first admission for rheumatoid arthritis between cohorts. SD, standard deviation.

Mean age (±SD) at first admission was lower in FSPs than the general population for OA (55.1 ± 12.64 FSP versus 59.9 ± 12.04 MPC; *P* < 0.001), joint replacement (64.5 ± 9.90 FSP versus 66.9 ± 11.20 MPC; *P* = 0.034) and meniscal injury (49.2 ± 7.6 FSP versus 51.2 ± 8.6 MPC; *P* = 0.001). In contrast, there was no difference in mean age at first admission for RA (Figure 2).

**Figure 3.**
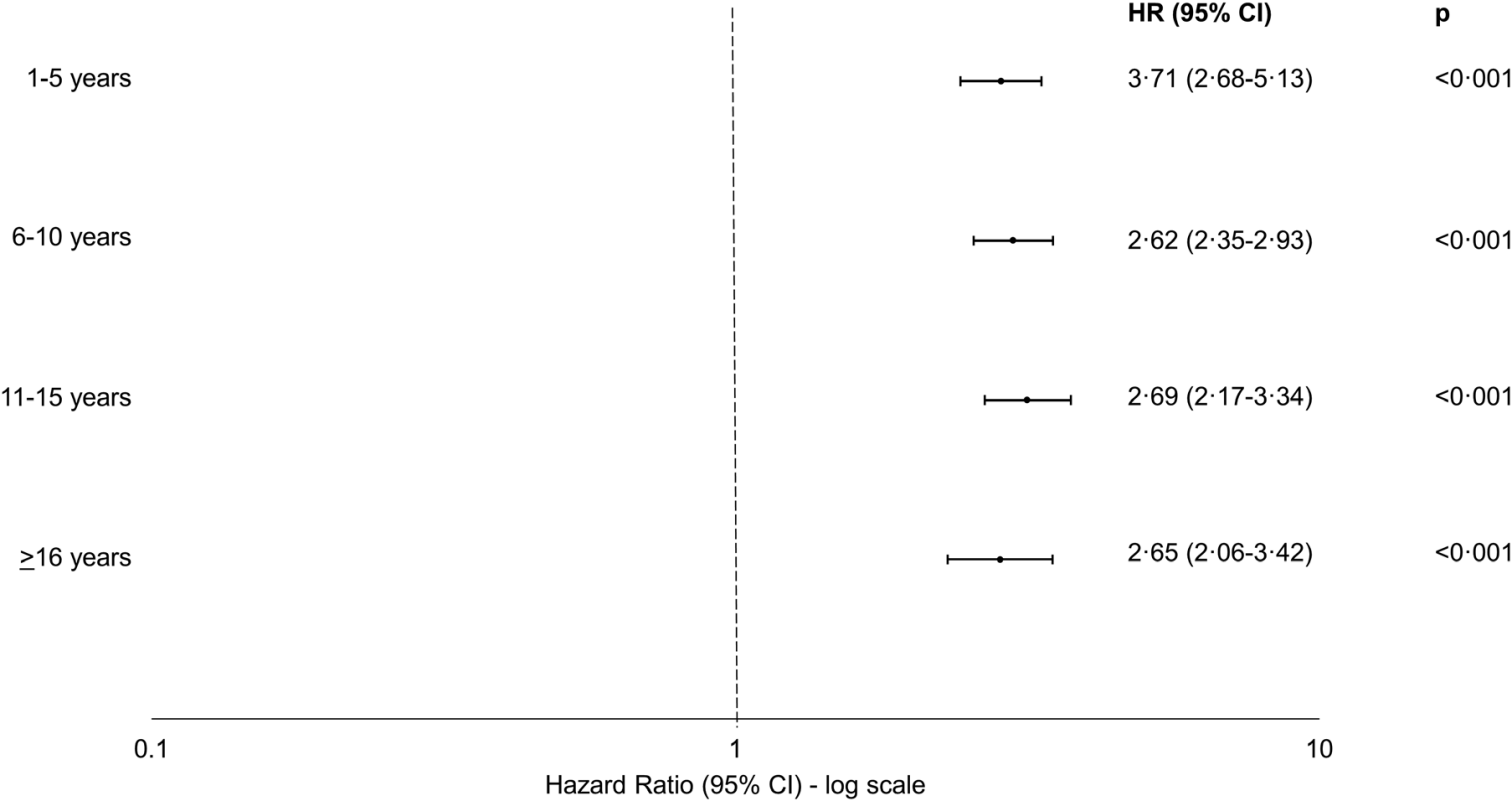
Career length and risk of osteoarthritis among male former professional soccer players compared with a matched general population control group. Compared with matched population controls, risk of osteoarthritis remained similar in former soccer players for all career length categories (*P* = 0.71, likelihood-ratio test).

**Figure 4.**
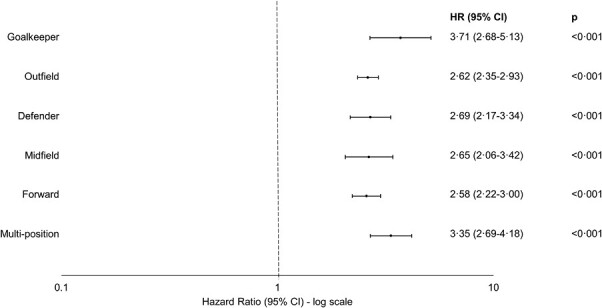
Player position and risk of osteoarthritis among male former professional soccer players compared with a matched general population control group. Compared with matched population controls, risk of osteoarthritis remained similar across all player positions (*P* = 0.14, likelihood-ratio test).

Compared with MPC, the risk of OA was similar across the 5-year bands of career duration (*P* = 0.71, likelihood-ratio test; Figure 3). Field position data were available for 6622 (86.3%) of the 7676 FSPs. Compared with MPC, the risk of hospital admission for OA was significantly higher for all player positions, with no significant difference in the strength of association by player position (*P* = 0.14, likelihood-ratio test; Figure 4).

## Discussion

Our findings demonstrate a higher risk of musculoskeletal disease, particularly hospital admission for lower limb OA, meniscal injury and joint replacement among former professional soccer players when compared with MPC.

The majority of prior research in this area highlights a potential increased risk of musculoskeletal problems, particularly of the lower limb, in soccer athletes, and our findings of increased OA in the lower limb, but not the upper limb, corroborate and build on this. In an Italian study, knee and hip arthroplasties were more frequent among male FSPs than a control group [25]. In a postal questionnaire study, knee OA was also reported more commonly by a cohort of FSPs compared to a sample of men from the general population (adjusted risk ratio: 3.73, 95% CI 3.33–4.17) [26]. However, given the response rate of 50%, there was potential for selection bias, with former players suffering musculoskeletal problems perhaps being more likely to respond to a questionnaire on the topic. This present study acts to minimize bias by utilizing medical records instead of self-reported data.

Results of this study show an age-dependent variability, with an increased risk of hospital admission for OA among FSPs being higher in younger individuals and falling at later ages. Post-traumatic OA has an earlier onset than traditional OA and is more common in younger, more active individuals [27]. Prior studies have shown that individuals with a history of early-onset OA also require surgical intervention at an earlier age [27].

It is of interest that our study has shown former professional soccer players are at an increased risk of meniscal injury when compared to matched controls. Meniscal injury and anterior cruciate ligament rupture are well-recognized risk factors for developing OA of the knee [28]. One study of female soccer players with a mean age of 31 years found that 82% of players had radiographic OA 12 years after an anterior cruciate ligament rupture, compared with 37% of uninjured knees [29].

One explanation for the increased overall risk and earlier age of first hospital admission, seen for both OA and joint replacement in our cohort of former professional soccer players, maybe prior knee joint injury (post-traumatic OA). There may be other factors influencing OA risk in elite-level athletes, with evidence suggesting excessive use of knee intra-articular injections, used to manage injury-related pain, may increase the risk of knee pain and OA in the long term [30]. Due to limitations in health record data, we were unable to gain access to such information in this cohort. We also acknowledge that we were unable to pinpoint the exact location on the body of the arthroplasty and/or OA in our cohort and were unable to stratify further than the upper or lower limb. However, since knee OA is more common than hip OA [31], it is plausible that our finding of increased risk of lower limb OA in former professional footballers is driven by knee OA. Furthermore, this study does not capture information on patients who do not require review by hospital services. However, we have no reason to presume that there would be a difference in diagnostic reporting or the level of healthcare accessed by former professional footballers and their matched controls.

OA risk was not shown to be associated with career length. It could be hypothesized that those enduring a longer career may also be exposed to greater injury risk, in which case, an association between career length and OA risk may be expected. Studies have highlighted soccer players may end their career as a direct result of OA-related pain and impairment problems [1], and in some cases, are forced to retire from the game early due to injury or pain, potentially explaining the absence of any association between OA risk and career length.

Trauma resulting from sports participation has been linked with the subsequent development of OA [32,33], which may be an underlying risk factor in this cohort. Currently, medical interventions are mostly symptomatic, and are inadequate to prevent the development of OA, or to dramatically improve pre-existing OA. The global governing body for football, The Fédération internationale de football association (FIFA), has developed a training programme which has been shown to reduce risk of injury in the lower limb in soccer athletes, and expansion of programmes such as this may reduce injury incidence and subsequent OA [34].

In this study, we also stratified players into their positional categories to assess whether certain positions known to have greater injury incidence, had associated elevated OA outcomes. Prior reports have stated that the position played has little effect on injuries, with injuries being evenly distributed across positions [35]. This current study shows no difference in risk of OA across positional categories.

For comparison purposes as a ‘negative control’, RA in former professional soccer players was also investigated. RA and OA can both cause joint pain and stiffness. Both are forms of arthritis but have different aetiology. The repetitive movements and overuse of joints as a result of a career in elite-level sport is a plausible mechanism for increased risk of OA in former professional soccer players, but would not predispose to RA. Therefore, the finding of an elevated risk in FSPs that was restricted to OA and not observed for RA is evidence in support of the former being likely due to trauma.

In their 2020 report, the Industrial Injuries Advisory Council (IIAC) stated that they did not find enough consistent evidence to suggest a doubling in OA risk among soccer players, leading to the conclusion that OA of the knee in soccer players was not considered an industrial injury [5]. The results of this current study, however, show around three times greater risk of any OA, and around three-and-a-half times greater risk of lower limb OA, among former professional soccer players when compared with general population controls. As knee OA is more common than hip OA [30], it is plausible that this finding is largely driven by knee OA. Further, while we see this increase in OA, rates of RA are no different between soccer players and their controls, providing evidence that the association with OA is unlikely to be a spurious finding.

This study showed an age-associated increase in risk of OA in FSPs when compared with MPC, which was attributed to lower limb OA. Associated with this were greater hospital admissions for knee meniscal injuries and joint replacement in the former professional soccer player cohort. In contrast, there was no increase in RA. These data add to the evidence base relating to risk of musculoskeletal issues in soccer players and add to data in support of lower limb osteoarthritis representing an industrial injury in these former athletes.

## Data Availability

Data available upon reasonable request and subject to appropriate approvals from National Health Service (NHS) Scotland’s Public Benefit and Privacy Panel for Health and Social Care.
